# Hormone-Like Effects of Bisphenol A on p53 and Estrogen Receptor Alpha in Breast Cancer Cells

**DOI:** 10.1089/biores.2018.0048

**Published:** 2019-10-31

**Authors:** Victoria Lloyd, Mia Morse, Betsy Purakal, Jordan Parker, Paige Benard, Michael Crone, Samantha Pfiffner, Monica Szmyd, Sumi Dinda

**Affiliations:** Department of Biomedical Diagnostic and Therapeutic Sciences, School of Health Sciences, Center of Biomedical Research, Oakland University, Rochester, Michigan.

**Keywords:** antiestrogens, BPA, breast cancer, ERα, estrogen, p53, tumor suppressors

## Abstract

Bisphenol A (BPA) is a polymerizing agent commonly found in plastics that has been linked to xenoestrogenic activity. In this study, we analyzed the estrogen-like effects of BPA on the expression of estrogen receptor (ER)α and p53 with hormonal and antihormonal treatments in T-47D and MCF-7 cells. Cells were cultured in medium containing 5% charcoal-stripped fetal bovine serum for 6 days to deplete any endogenous steroids or effectors. The cells were then treated for 24 h with 600 nM BPA, which was determined to be the optimal value by a concentration study of BPA from 1 nM to 2 μM. Extracted cellular proteins were quantified and subjected to sodium dodecyl sulfate–polyacrylamide gel electrophoresis (SDS-PAGE)/Western blot analysis. The cell proliferation assays were quantified upon exposure to BPA. Laser confocal microscopy was performed to determine the cytolocalization of p53 and ERα upon treatment with BPA. Western blot analysis revealed that BPA caused an increase in the cellular protein p53 in a concentration-dependent manner. While treatment with BPA did not affect the cytolocalization of p53, an increase in cell proliferation was observed. Our studies provide interesting leads to delineate the possible mechanistic relationship among BPA, ER, and tumor suppressor proteins in breast cancer cells.

## Introduction

The normal growth of cells and cellular proliferation are highly regulated processes. The structural and functional changes in the human breast are influenced by the activation and inactivation of oncogenes or mutations in tumor suppressor genes such as p53 and retinoblastoma protein (pRb). In a normal state, p53 works to regulate the cell cycle at the G1 checkpoint, preventing any damaged DNA from progressing into the S phase. In response to cellular stress, p53 is activated through phosphorylation.^1^ Similarly, pRb also works at the G1 checkpoint. In its hypophosphorylated and active form, pRb is bound to the E2F transcription factor, repressing transcription of proteins needed for cell division. When further phosphorylated, pRb detaches from E2F, thus allowing transcription of proteins needed for DNA replication in the S phase.^2,3^ Inactivated p53 and pRb can promote the transformation of a normal cell into a malignant cancer cell. When these tumor suppressor proteins are inactive, cells can undergo uncontrolled proliferation, leading to worse prognoses for breast cancer patients.^2,3^

The quantitative presence of estrogen receptors (ERs) in breast cancer cells is indicative of their ER positive or ER negative classification.^4^ Much of the therapy surrounding breast cancer depends on the existence of ERs on the malignant cells. In ER-positive cases, 17β-Estradiol (E_2_) has the ability to bind to the ER present within malignant cells and induce further proliferation. The T-47D breast cancer cell line is ER and progesterone receptor positive. In addition, this cell line expresses p53 with a missense mutation at codon 194, thus converting leucine to phenylalanine.^5^ Our previous studies have shown that E_2_ causes a significant increase in expression of p53 in the T-47D human breast carcinoma cell line. This suggests that E_2_ stimulates proliferation of the T-47D cell line.^6^ The MCF-7 breast cancer cell line is also ER positive. It is sensitive to hormones through the expression of ER, which makes it suitable for our studies. Therapies using selective ER modulators use compounds that act as ER antagonists or agonists based on the tissue specificity.^7^ The ER antagonists such as ICI 164,384 and Tamoxifen (TAM) can bind to the ER and hinder E_2_ binding, thus blocking these receptor-mediated effects and inhibiting breast cancer cell proliferation.^7^

Since the 1960s, the organic compound bisphenol A (BPA) became widely used in the United States manufacturing industry in the production of polycarbonate plastics and epoxy resins.^8^ Today, BPA is one of the highest-volume chemicals currently in production.^9^ Concerns about increased human exposure to BPA became relevant due to its widespread use in beverage containers, dental sealants, and other plastic products.^8^ Further studies came out showing that, at high temperatures, BPA leaches out from lacquer-coated cans and is present as a contaminant in the foods stored in those cans.^10^ One study reported that BPA could be detected in bodily fluids of more than 90% of the human population.^9^ Another study found evidence of BPA in the urine samples of over 95% of the sample population tested (394 humans).^11^ Multiple reports have also shown that BPA is present in humans at nanomolar concentrations and that it is, indeed, effective at such concentrations.^11–15^

Since its introduction into the industry, BPA has been shown to have estrogen-like qualities.^8^ Researchers have also demonstrated that BPA produces estrogenic actions within the body, and its effects are similar to that of E_2_.^8^ Recent studies have shown that, like E_2_, BPA has the ability to cause changes in cellular function at concentrations between 1 pM and 1 nM. The amounts of BPA measured in maternal and fetal blood far exceed these values.^10^ Several studies have indicated that environmentally relevant concentrations of BPA act additively with ovarian estrogens.^16,17^The estrogenic activity of BPA has led to concerns about its ability to cause breast cancer in humans. Recent studies conducted using mice and rats have shown evidence that there is a correlation between BPA exposure and the development of breast cancer. In addition, studies have also shown that BPA may potentially interfere with breast cancer therapy.^16^ Numerous studies have shown that rats exposed early in life to BPA have an increased susceptibility to mammary tumorigenesis.^18,19^ Several studies conducted in rodents raise concern that exposure to low doses of BPA may have deleterious effects in various hormone-responsive organs.^9,20,21^ Furthermore, additional studies have suggested that BPA has the ability to inactivate tumor suppressor genes and thus increase the likelihood of tumor formation.^22^

Our laboratory studies have focused on the hormonal regulation of tumor suppressor proteins in breast cancer cells. We have previously shown that treatment of T-47D breast cancer cells with 17β-Estradiol (E_2_) causes cell proliferation, upregulates tumor suppressor protein p53, and induces hyperphosphorylation of pRb.^6,7^ The focus of the present study was to investigate the estrogen-like effects of BPA on T-47D and MCF-7 breast cancer cells and determine if these ER-mediated effects of BPA are inhibited in the presence of antiestrogens, such as ICI 164,384, Raloxifene (RAL), and TAM. If BPA does indeed affect such an essential tumor suppressor protein as p53, then such findings will be crucial in evaluating the treatment and prevention of breast cancer.

## Materials and Methods

TAM and ICI 164,384 were purchased from Tocris Bioscience (Ellisville, MO). Anti-pRb (G3-245) and anti-p53 (p53/80) monoclonal antibodies were purchased from BD Biosciences (San Jose, CA). Estradiol (E_2_), BPA, insulin, and activated charcoal were ordered from Sigma (St Louis, MO). RPMI 1640, antibiotic antimycotic solution, l-glutamine, and HEPES were purchased from Hyclone (Logan, UT). Radioimmunoprecipitation assay (RIPA) lysis buffer, goat anti-mouse secondary antibody, and protease inhibitors were purchased from Santa Cruz Laboratories (Santa Cruz, CA).

T-47D cells were obtained from American Type Culture Collection. Cells were routinely cultured at 37°C in an incubator in RPMI 1640 media with l-glutamine supplemented with 5% heat inactivated fetal bovine serum (FBS) or single stripped FBS (SSFBS), 25 mM HEPES, 24 mM sodium bicarbonate, 0.5% 100 X nonessential amino acids, 100 U/mL penicillin, 0.1 mg/mL streptomycin, 0.25 μg/mL amphotericin B, and 0.14 IU/mL insulin. MCF-7 cells were supplied by Corning Cellgro and cultured in an incubator at 37°C in Eagle's minimum essential medium (MEM) with 5% heat inactivated FBS or SSFBS, 1.5 g/L sodium bicarbonate, nonessential amino acids, l-glutamine, and sodium pyruvate. Stock solutions of the ligand were prepared in ethanol to a 1000-fold higher concentration than the final concentrations used during cell treatment. Two microliters of ligand were added directly to 2 mL of media. For proliferation studies, cells were passed into triplicate Corning 12-well dishes. After treatments with BPA, estrogen (E_2_), or anti-estrogen, cell number was determined by a Coulter counter model Z2. For cellular viability studies, cells were cultured in triplicate 12-well dishes and treated with various ligands. The live versus total cells in a population were determined using Nexcelom Cellometer Vision.

### Extraction of cells

Media was removed by aspiration; the cells were washed with 10 mL of Hanks balanced salt solution and scraped into 200 μL of extraction buffer (RIPA buffer, 1 mM leupeptin, 30 μg/mL pepstatin-A, 30 μg/mL chymostatin 0.3 mM PMSF). Cells were thawed on ice. A high-speed supernatant (HSS) of the extract was prepared using centrifugation of the burst cells at 15,000 RPM for 15 min at 4°C. The HSS was frozen in liquid nitrogen and stored at −80°C until further use.

### Sodium dodecyl sulfate–polyacrylamide gel electrophoresis and Western blot analysis

HSSs were denatured, and a protein assay was performed using the Bradford Method to standardize the amount of protein being loaded into each lane. Thirty micrograms of total protein/lane was resolved on Bio-Rad TGX mini gels under denaturing conditions. Proteins were transferred to Immobilon PVDF membrane (Millipore) at 100 V for 1 h in a tris-glycine buffer system containing 0.025% sodium dodecyl sulfate (SDS) and 15% methanol using a Bio-Rad Trans-Blot cell with tap water cooling. Membranes were blocked for 1 h in tris-buffered saline (TBS)-Tween (0.1%) plus 5% Carnation instant nonfat dry milk (NFDM) and then probed for 1 h in the same solution containing a 1:1000 dilution of primary antibodies, either anti-p53 monoclonal antibody (Transduction Laboratories) or anti-ERα monoclonal antibody (Santa Cruz Biotechnology). The membrane was then washed for 30 min with three changes of TBS-Tween, reblocked for 30 min with TBS-Tween plus 5% Carnation instant NFDM, and then incubated in the same solution plus a 1:1000 dilution of HRP-conjugated anti goat-mouse IgG H + L chain (Boehringer Mannheim). Actin bands were subsequently visualized utilizing anti-actin monocolonal antibody clone 4 (Millipore, Bedford, MA) and following the manufacturer's protocol. The specific band for each protein of interest was then visualized by the enhanced chemiluminescence method according to the instructions from Amersham (GE Healthcare Biosciences, Piscataway, NJ). The protein bands were visualized using the ChemiDoc XRS+ imaging system (Bio-Rad). The Western blots were subjected to quantification of the protein band density using the Image Studio Lite program, version 3.1 (LI-COR Biosciences, Lincoln, NE).

### Cell viability

T-47D cells (30,000/well) were plated for 2 days in media containing whole FBS and then withdrawn from hormonal and other growth factor stimuli by culturing for 6 days in media containing stripped serum and the ligands. Fresh media and the ligands were replaced at 2-day intervals. After a 7-day incubation with E_2_, BPA, and combinations of E_2_ + BPA, the cell number was determined by a Coulter Counter.

MCF-7 cell viability assays were used to show the total number of live cells following a 6-day treatment of ligands at varying concentrations. The same protocol was followed as from previous studies in our laboratory.^17–19^ Studies were performed using triplicate 12-well culture plates with an initial cell count of 3.0 × 10^4^ cells per well. The cells were maintained in 1 mL culture medium containing 10% FBS for 2 days. For the next 6 days, growth factor media was replenished with dextran-coated charcoal (DCC)-FBS media and treated with ligands over 2-day intervals for 6 days. Cells were treated with 600 nM BPA alone and in combination with E_2_, ICI, TAM, and RAL, followed by performance of a cellular viability assay. The cells were trypsinized, extracted from their wells, stained with propidium iodide, and underwent image cytometry using the Cellometer Vision CBA software (Nexcelom Bioscience LLC, Lawrence, MA).

### Immunofluorescence and confocal microscopy

T-47D cells were plated on cover-slips in 12-well plates (30,000 cells/well) and cultured for 48 h in 10% FBS. The cell culture medium was then changed to 5% DCC-FBS, and fresh medium was added at 2-day intervals. The cells were cultured in 5% DCC-FBS for a total of 6 days. On day 6, the ligands were suspended in 5% DCC-FBS media, and semiconfluent cells were treated for 24 h.

Immunolabeling was performed as previously described for p53.^7,23^ Briefly, the cells were fixed on cover-slips with 1% formalin in phosphate-buffered saline (PBS), permeabilized with ice-cold acetone and methanol (50:50), and washed thrice with PBS. Staining procedures were performed in a humidified chamber at 25°C. Cells were incubated in 10% goat serum (Sigma) to suppress nonspecific binding of IgG, followed by 2 h incubation with 1:200 dilution anti-p53 monoclonal antibody. The cells were then incubated for 3 h with 1:200 dilution anti-mouse IgG conjugated with Cy3 (Jackson ImmunoResearch Laboratories, West Grove, PA). Cover-slips were washed in PBS and incubated for 2 min in 1 μg/mL 4′,6-diamidino-2-phenylindole (DAPI) dissolved in PBS. Cells were washed in PBS, mounted with Fluoromount-G (Electron Microscopy Sciences, Hatfield, PA), and stored in the dark at 4°C.

The distribution of three-dimensional fluorescent structures was analyzed using a Nikon Digital Eclipse C1 plus confocal microscope, and differential interference contrast images were taken in parallel. NIS Elements AR software (Nikon Instruments, Melville, NY) was used for image analysis.

## Results

### Concentration-dependent effects of BPA on p53 and ERα levels

**FIG. 1.**
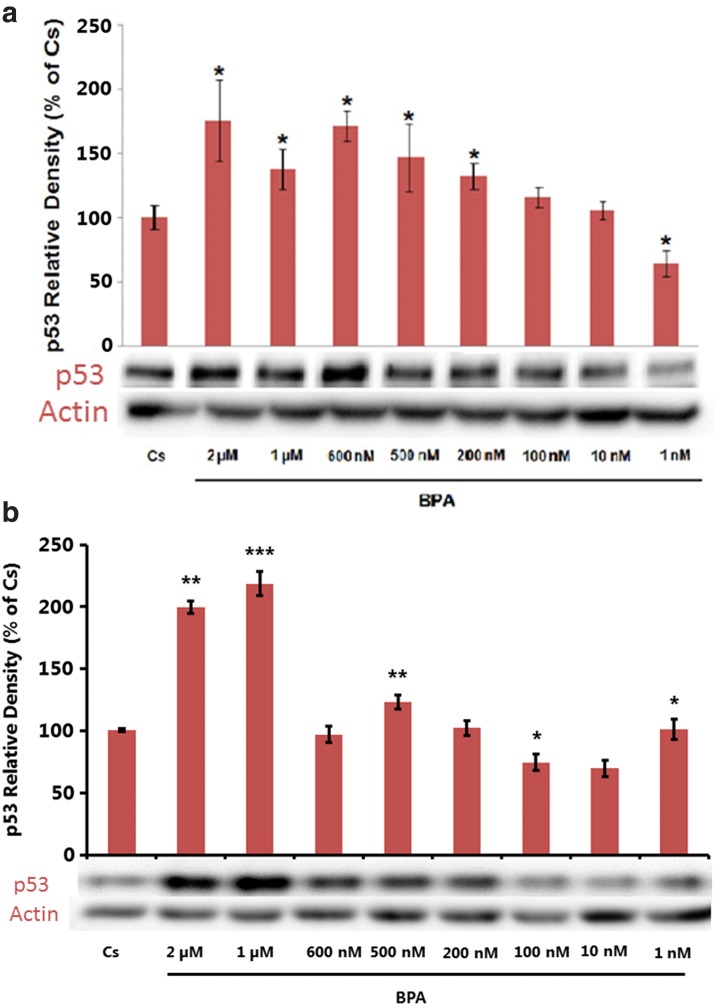
**(a)** T-47D cells were grown for 2 days in RPMI-1640 medium supplemented with 10% FBS and then cultured for 6 days in medium containing 5% DCC-stripped FBS. Semiconfluent cells were then treated with varying concentrations of BPA (1 nM–2 μM). Cellular extracts were prepared and subjected to protein quantification, SDS-PAGE, and Western blot analysis. Lanes labeled Cs represent control lanes with cells grown in the absence of ligands in medium containing 5% SS FBS. Actin bands are shown to indicate equal protein loading among treatment conditions. The relative intensity of p53, compared to Cs, is displayed as the mean ± SEM. The sample sizes came from five density measurements per group. The asterisk indicates significant difference with the control at *p* < 0.05 (Kruskal–Wallis test followed by *post hoc* analysis using Mann–Whitney *U*-test). Representative Western blots are shown. **(b)** MCF-7 cells were grown for 2 days in Eagle's MEM and supplemented with 10% FBS and then cultured for 6 days in medium containing 5% DCC-stripped FBS. Semiconfluent cells were then treated with varying concentrations of BPA (1 nM–2 μM). Cellular extracts were prepared and subjected to protein quantification, SDS-PAGE, and Western blot analysis. Lanes labeled Cs represent control lanes with cells grown in the absence of ligands in medium containing 5% SS FBS. Actin bands are shown to indicate equal protein loading among treatment conditions. The relative intensity of p53, compared to Cs, is displayed as the mean ± SEM. The sample sizes came from five density measurements per group. The asterisk indicates significant difference with the control at *p* < 0.05 (Kruskal–Wallis test followed by *post hoc* analysis using Mann–Whitney *U*-test). Representative Western blots are shown. BPA, bisphenol A; DCC, dextran-coated charcoal; SS, stripped serum; FBS, fetal bovine serum; MEM, minimum essential medium; SDS-PAGE, sodium dodecyl sulfate–polyacrylamide gel electrophoresis; SEM, standard error of the mean.

Figure 1a demonstrates the p53 expression profiles of control and BPA-treated T-47D cells cultured for 2 days in media containing whole FBS and then 6 days in media containing stripped serum. On the sixth day, cells were treated with BPA at concentrations ranging from 1 nM to 2 μM. Figure 1b displays the same studies performed in MCF-7 cells for comparison. Actin, a housekeeping protein consistently expressed under both normal and treatment conditions, was probed as a control to confirm equivalence in protein loading among wells.

We monitored the cells' level of p53 expression after treatment with varying concentrations of BPA to conduct a more direct assessment of the compound's effect within the cells. In the control group (Cs), cells were cultured in stripped serum for 6 days and not exposed to BPA. In comparison to the control, Figure 1a demonstrates that BPA-treated T-47D cells showed increased expression of p53 in a concentration dependent manner from 1 nM to 2 μM. The optimal increase in p53 expression occurred when cells were treated with 600 nM of BPA. Figure 1b shows that BPA-treated MCF-7 cells also display an increase in p53 expression from 1 nM to 2 μM, but with slight variation from the studies done in T-47D cells. Our findings agree with other studies indicating that BPA demonstrates a concentration-dependent effect on p53 expression.^14^

**FIG. 2.**
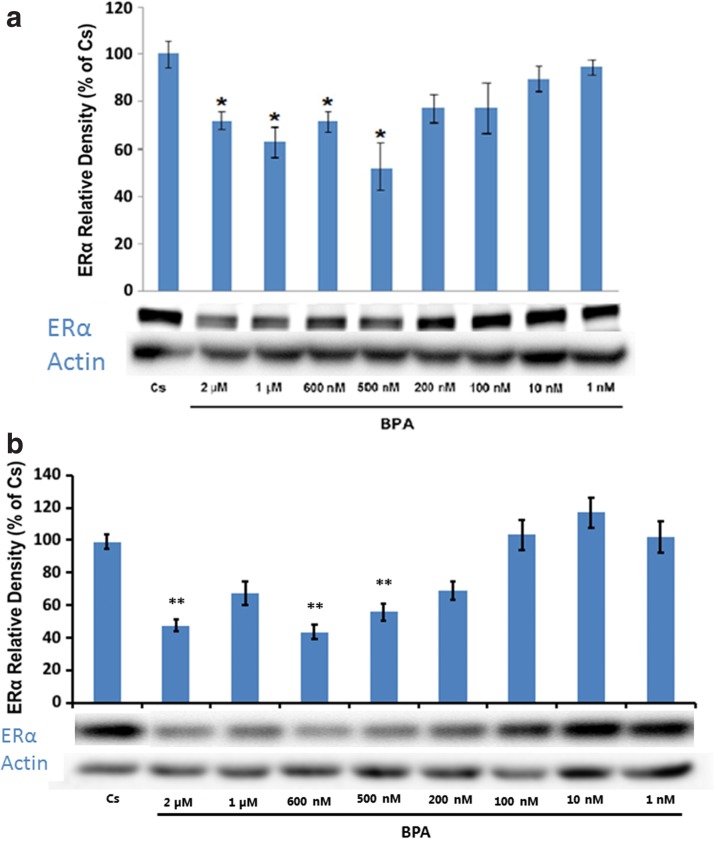
**(a)** T-47D cells were grown for 2 days in RPMI-1640 medium supplemented with 10% FBS and then cultured for 6 days in medium containing 5% DCC-stripped FBS. Semiconfluent cells were then treated with varying concentrations of BPA (1 nM–2 μM). Cellular extracts were prepared and subjected to protein quantification, SDS-PAGE, and Western blot analysis. Lanes labeled Cs represent control lanes with cells grown in the absence of ligands in medium containing 5% SS FBS. Actin bands are shown to indicate equal protein loading among treatment conditions. The relative intensity of ERα, compared to Cs, is displayed as the mean ± SEM. The sample sizes came from five density measurements per group. The asterisk indicates significant difference with the control at *p* < 0.05 (Kruskal–Wallis test followed by *post hoc* analysis using Mann–Whitney *U*-test). Representative Western blots are shown. **(b)** MCF-7 cells were grown for 2 days in Eagle's MEM supplemented with 10% FBS and then cultured for 6 days in medium containing 5% DCC-stripped FBS. Semiconfluent cells were then treated with varying concentrations of BPA (1 nM–2 μM). Cellular extracts were prepared and subjected to protein quantification, SDS-PAGE, and Western blot analysis. Lanes labeled Cs represent control lanes with cells grown in the absence of ligands in medium containing 5% SS FBS. Actin bands are shown to indicate equal protein loading among treatment conditions. The relative intensity of ERα, compared to Cs, is displayed as the mean ± SEM. The sample sizes came from five density measurements per group. The asterisk indicates significant difference with the control at *p* < 0.05 (Kruskal–Wallis test followed by *post hoc* analysis using Mann–Whitney *U*-test). Representative Western blots are shown.

The results in Figures 2a and b show the concentration-dependent effects of 1 nM–2 μM concentrations of BPA on the expression of ERα in T-47D cells and MCF-7 cells, respectively. In comparison to the control, BPA shows a downregulation of ERα in a concentration-dependent manner from 1 nM to 2 μM in both cell lines.

### Time course trials in T-47D cells

**FIG. 3.**
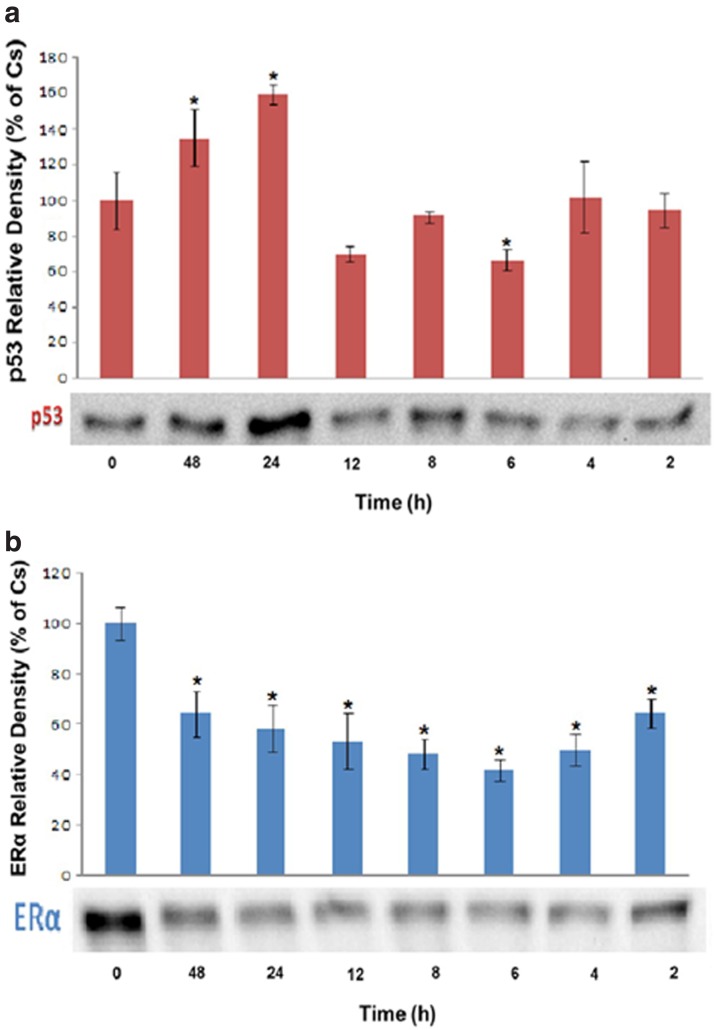
**(a)**: Cultures of T-47D cells with BPA were performed as described in Figures 1a and 2a. Cells were then treated with 600 nM BPA for the various time intervals indicated. Cellular extracts were prepared and subjected to protein quantification, SDS-PAGE, and Western blot analyses. The relative intensity of p53, compared to Cs, is displayed as the mean ± SEM. The sample sizes came from five density measurements per group. The asterisk indicates significant difference with the control at *p* < 0.05 (Kruskal–Wallis test followed by *post hoc* analysis using Mann–Whitney *U*-test). Representative Western blots are shown. **(b)** Cultures of T-47D cells with BPA were performed as described in Figures 1a and 2a. Cells were then treated with 600 nM BPA for the various time intervals indicated. Cellular extracts were prepared and subjected to protein quantification, SDS-PAGE, and Western blot analyses. The relative intensity of ERα, compared to Cs, is displayed as the mean ± SEM. The sample sizes came from five density measurements per group. The asterisk indicates significant difference with the control at *p* < 0.05 (Kruskal–Wallis test followed by *post hoc* analysis using Mann–Whitney *U*-test). Representative Western blots are shown.

A time course trial was performed in T-47D cells to further determine the efficiency with which BPA altered p53 expression. Cultures were prepared as described above. Cells were treated with 600 nM BPA (determined from Fig. 1 to be the most effective BPA concentration) from between 2 and 48 h before extraction. Cellular extracts were prepared and subjected to protein quantification using the Bradford Method, SDS-polyacrylamide gel electrophoresis (PAGE), and Western blot analysis. Figure 3a demonstrates that BPA induced a detectable and maximum increase in p53 levels when cells were treated for 24 h in comparison to the control. Figure 3b shows the results obtained after conducting a time course trial (0–48 h) that examined the effects of 600 nM BPA on the expression of ERα. The results in Figure 3b show that BPA induced a maximum decrease in ERα levels at 48 h in comparison to the control.

### Effect of BPA and ER antagonists on p53 and ERα levels in T-47D and MCF-7 cells

The primary focus of this study was to further understand the mechanisms of BPA action within the cells. Cells were treated with different combinations of either E_2_ or BPA and other ER antagonists, including ICI, RAL, and TAM, for 6 days in DCC-FBS stripped serum. Trace amounts of endogenous steroids can be removed through treatment of the cells with DCC-FBS medium.^23^ On the seventh day, cells were extracted, protein levels were quantified using the Bradford Method, and SDS-PAGE and Western blot analyses were performed.

**FIG. 4.**
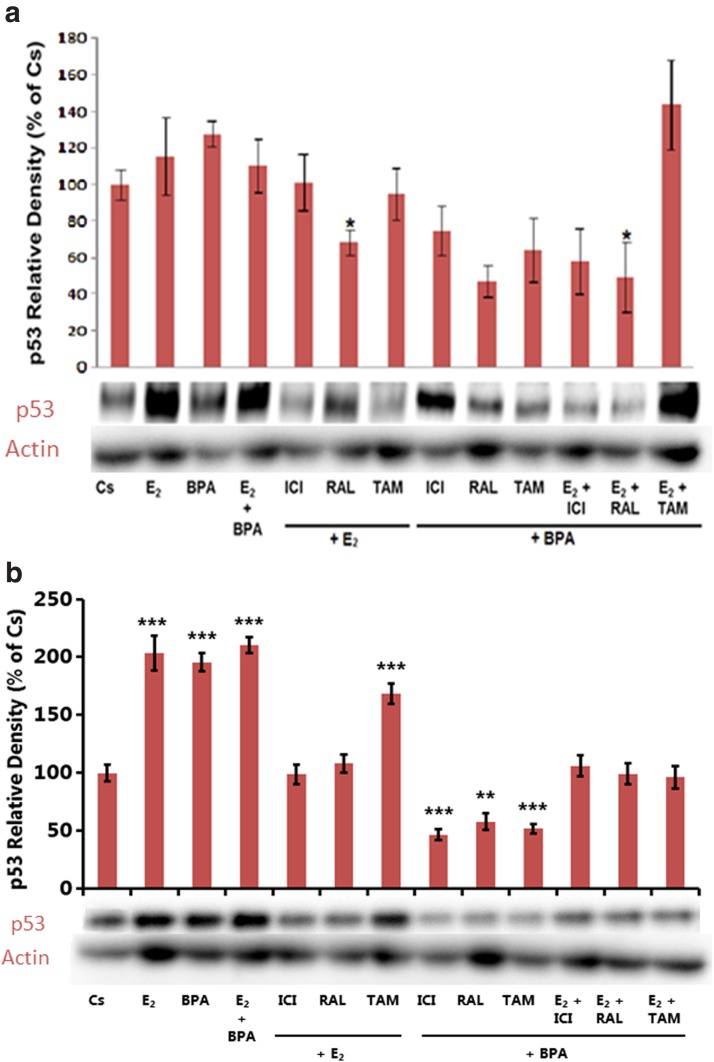
**(a)** Culture and treatment of T-47D cells were performed as described in Figures 1a and 2a. Cells were treated for 24 h with BPA (600 nM), E_2_ (1 nM), ICI (1 μM), TAM (1 μM), and RAL (1 μM). Cellular extracts were prepared and subjected to protein quantification, SDS-PAGE, and Western blot analysis. Actin bands are shown to indicate equal protein loading among treatment conditions. The relative intensity of p53, compared to Cs, is displayed as the mean ± SEM. The sample sizes came from five density measurements per group. The asterisk indicates significant difference with the control at *p* < 0.05 (Kruskal–Wallis test followed by *post hoc* analysis using Mann–Whitney *U*-test). Representative Western blots are shown. **(b)** Culture and treatment of MCF-7 cells were performed as described in Figures 1b and 2b. Cells were treated for 24 h with BPA (600 nM), E_2_ (1 nM), ICI (1 μM), TAM (1 μM), and RAL (1 μM). Cellular extracts were prepared and subjected to protein quantification, SDS-PAGE, and Western blot analysis. Actin bands are shown to indicate equal protein loading among treatment conditions. The relative intensity of p53, compared to Cs, is displayed as the mean ± SEM. The sample sizes came from five density measurements per group. The asterisk indicates significant difference with the control at *p* < 0.05 (Kruskal–Wallis test followed by *post hoc* analysis using the Mann–Whitney *U*-test). Representative Western blots are shown. E_2_, 17β-estradiol; RAL, raloxifene; TAM, tamoxifen.

Figure 4a demonstrates the resulting p53 levels in each sample of T-47D cells. The highest expression of p53 was observed when cells were treated with E_2_ + TAM and E_2_ alone. This was expected, as our previous studies have indicated that estrogen largely increased the expression of p53 levels.^1^ Our laboratory has also previously demonstrated that treatment with physiological concentrations of E_2_ (0.1–1 nM) causes an increase in the expression of p53.^6,7^ As expected, when cells were treated with E_2_ and the complete ER antagonist ICI, the increase in p53 levels was reversed back completely to that of the control. When cells were treated with BPA alone, there was a slight increase in p53 expression. However, when treatment combined BPA and ICI, p53 expression decreased in a manner similar to that of the E_2_ and ICI treatment. Combination treatment RAL with E_2_ and BPA had decreased the p53 expression, which was similar to the ICI treatment.

Figure 4b displays the resulting p53 levels in response to various treatments in MCF-7 cells. Compared to the control, treatment with BPA alone depicts a 96% increase in p53 expression, and E_2_ alone showed a 104% increase. When treatments of E_2_ and ICI were combined, p53 expression was decreased compared to E_2_ alone. Treatment of BPA + ICI showed a significant decrease in p53 expression compared to BPA alone, which suggests that the ER antagonist, ICI, is also acting antagonistically to BPA.

**FIG. 5.**
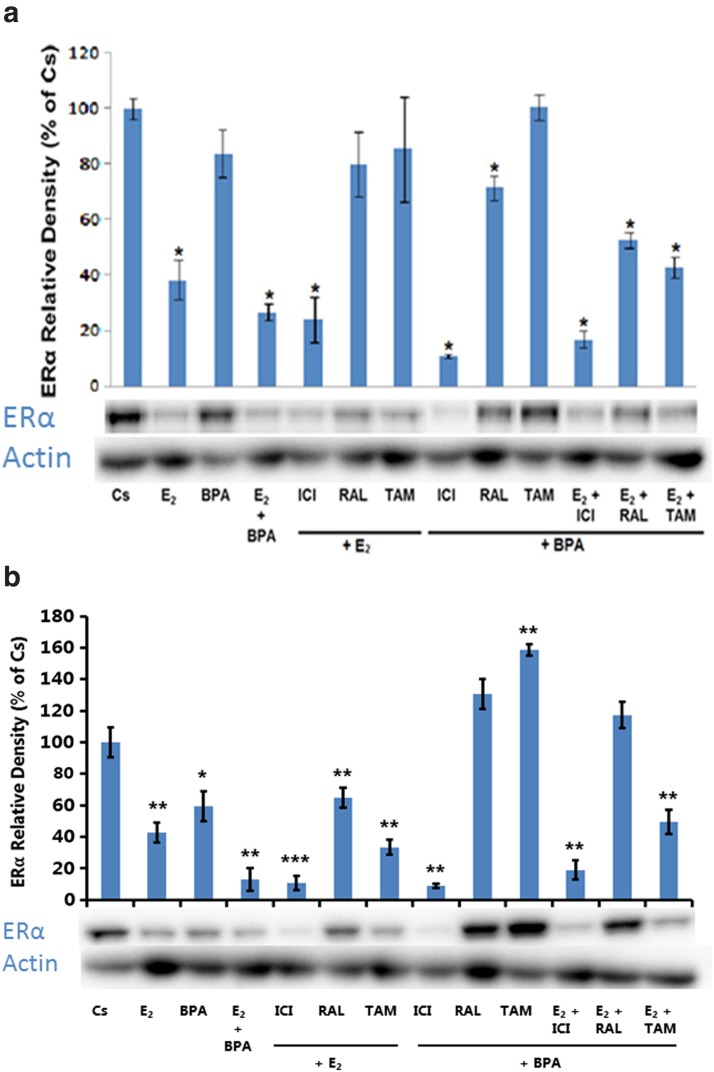
**(a)** Culture and treatment of T-47D cells were performed as described in Figures 1a and 2a. Cells were treated for 24 h with BPA (600 nM), E_2_ (1 nM), ICI (1 μM), TAM (1 μM), and RAL (1 μM). Cellular extracts were prepared and subjected to protein quantification, SDS-PAGE, and Western blot analysis. Actin bands are shown to indicate equal protein loading among treatment conditions. The relative intensity of ERα, compared to Cs, is displayed as the mean ± SEM. The sample sizes came from five density measurements per group. The asterisk indicates significant difference with the control at *p* < 0.05 (Kruskal–Wallis test followed by *post hoc* analysis using Mann–Whitney *U*-test). Representative Western blots are shown. **(b)** Culture and treatment of MCF-7 cells were performed as described in Figures 1b and 2b. Cells were treated for 24 h with BPA (600 nM), E_2_ (1 nM), ICI (1 μM), TAM (1 μM), and RAL (1 μM). Cellular extracts were prepared and subjected to protein quantification, SDS-PAGE, and Western blot analysis. Actin bands are shown to indicate equal protein loading among treatment conditions. The relative intensity of ERα, compared to Cs, is displayed as the mean ± SEM. The sample sizes came from five density measurements per group. The asterisk indicates significant difference with the control at *p* < 0.05 (Kruskal–Wallis test followed by *post hoc* analysis using Mann–Whitney *U*-test). Representative Western blots are shown.

Figure 5a shows the level of ERα expression in T-47D cells upon treatment with BPA, hormones, and antihormones. Treatment with BPA alone or the concomitant treatment of E_2_ + BPA resulted in a downregulation of ERα compared to the control. Figure 5b presents the level of ERα expression in MCF-7 cells after treatment with BPA, hormones, and antihormones. As with T-47D cells, treatment with BPA and BPA + E_2_ also resulted in a downregulation of ERα compared to the control, showing that ERα expression is consistent across both cell lines following treatments with the various ER agonists and antagonists used in this study.

### BPA-induced proliferation of cell count

T-47D cells (30,000/well) were plated in triplicates in 12-well plates in media (FBS) containing growth factors for 2 days. Fresh DCC-FBS media and ligands were replaced every 48 h. After a 7-day incubation with E_2_, BPA, ICI, and combinations of E_2_ + BPA, BPA + ICI, and BPA + TAM, the cell number was determined by a Coulter Counter.

**FIG. 6.**
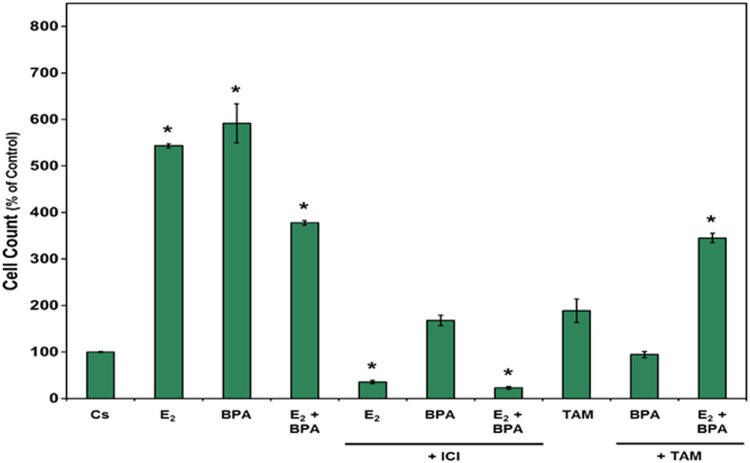
Cells were grown in triplicates in 12-well plates with 30,000 cells per well in media (FBS) containing growth factors for 2 days. Fresh charcoal media and ligands were replaced in 2-day intervals. On the seventh day, cell proliferation count was determined using the Coulter Counter. The relative intensities are, compared to Cs, displayed as the mean ± SEM. The sample sizes came from five density measurements per group. The asterisk indicates significant difference with the control at *p* < 0.05 (Kruskal–Wallis test followed by *post hoc* analysis using Mann–Whitney *U*-test).

Figure 6 illustrates the ability of BPA to induce proliferation in T-47D cells. In comparison to the control, both BPA and E_2_ induced increased levels of cell proliferation, with cell counts between five and six times higher than the control group. Combination treatment of ICI and BPA + E_2_ inhibited cell proliferation to similar degrees compared to the individual treatment of E_2_ and BPA. TAM, in combination with BPA, showed less proliferation than BPA combined with ICI.

### Effects of BPA, hormones, and antihormones on cellular viability

**FIG. 7.**
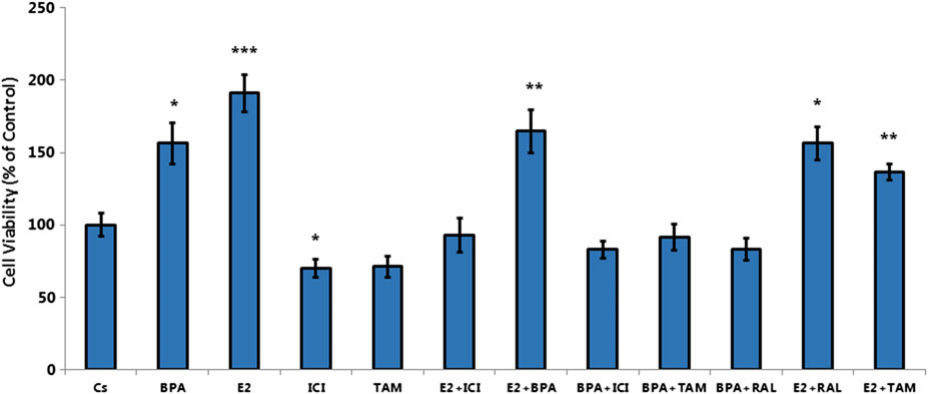
MCF-7 cells were cultured in 12-well plates containing 30,000 cells per well. Cells were grown for 2 days in 10% FBS media containing growth factors. For the following 6 days, growth factor media was replenished with DCC-FBS media and treated with ligands at 2-day intervals over 6 days. The treatments consisted of various combinations of BPA (600 nM), E_2_ (1 nM), ICI (1 μM), TAM (1 μM), and RAL (1 μM). A cellular viability assay was performed on the seventh day utilizing propidium iodide staining and image cytometry using the Nexcelom Cellometer Vision. Single asterisks are indicative of significant difference with comparison to the control at *p* < 0.05 and ***p < 0.001 (Kruskal–Wallis test followed by *post hoc* analysis using Mann–Whitney *U* test). Three independent experiments are displayed in the graph.

MCF-7 cell viability was examined after various treatment combinations of BPA, hormones, and antihormones. In Figure 7, treatments of E_2_, BPA, and E_2_ + BPA display a similar increase in cell viability compared to the control, further demonstrating the estrogenic properties of BPA in MCF-7 cells. Treatment of BPA and ICI combined displayed a significant decrease in cellular viability compared to the treatment with BPA alone, showing the antagonistic properties of ICI toward BPA.

### Effects of BPA, E2, and ICI on the immunolocalization of p53 in T-47D and MCF-7 cells

**FIG. 8.**
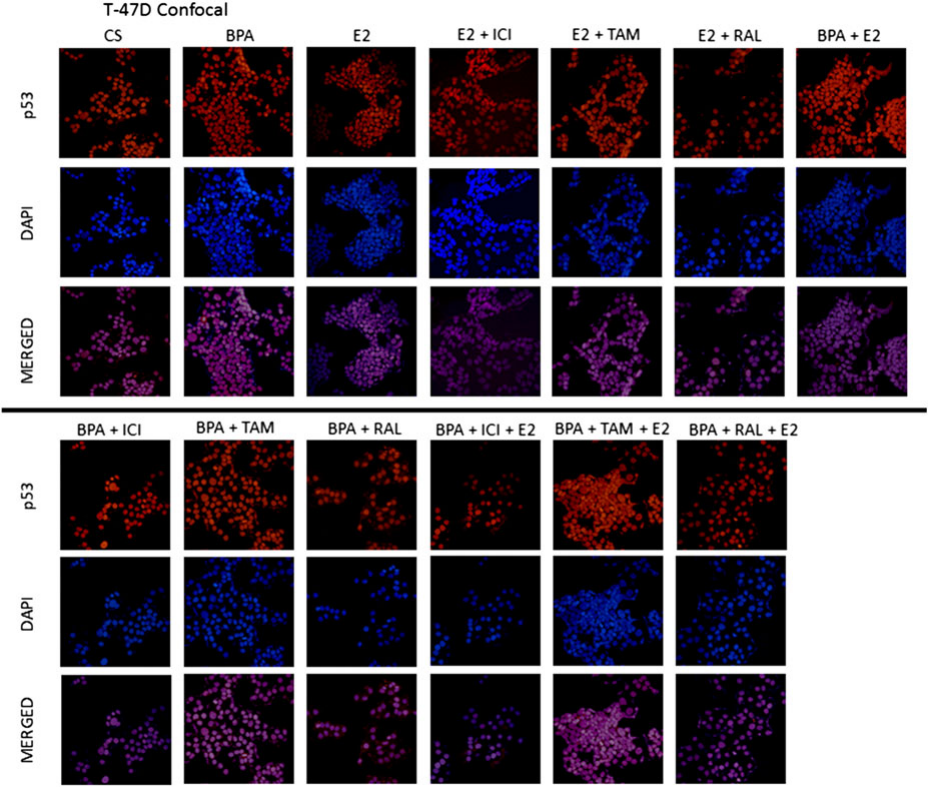
Treated T-47D cells were grown in 12-well growth plates, each well contained ∼30,000 cells on cover-slips. The cells were nourished for 2 days in whole media containing 10% FBS. They were then withdrawn from endogenous growth factors by culturing in DCC-FBS for 6 days. E_2_, BPA, ICI, RAL, and TAM were added in 2-day intervals for a period of 6 days. Cells were treated with Cy3 (red) and DAPI (blue) immunofluorescent stains, and the cytolocalization of p53 was determined using confocal microscopy. From the confocal microscopic images it is determined that p53 is located within the nuclei of T47D cells in all of the conditions. DAPI, 4′,6-diamidino-2-phenylindole.

**FIG. 9.**
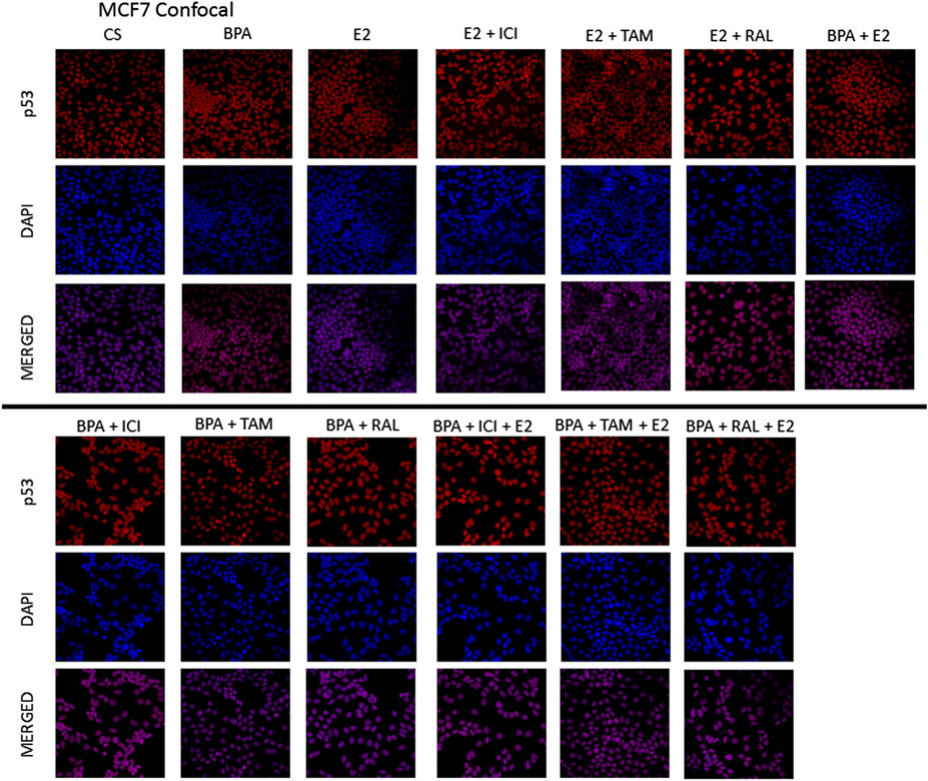
Treated MCF-7 cells were grown in 12-well growth plates, each well contained ∼30,000 cells on cover-slips. The cells were nourished for 2 days in whole media containing 10% FBS. They were then withdrawn from endogenous growth factors by culturing in DCC-FBS for 6 days. E_2_, BPA, ICI, RAL, and TAM were added in 2-day intervals for a period of 6 days. Cells were treated with Cy3 (red) and DAPI (blue) immunofluorescent stains, and the cytolocalization of p53 was determined using confocal microscopy. From the confocal microscopic images it is determined that p53 is located within the nuclei of MCF-7 cells in all of the conditions.

To determine if BPA's effect on the level of p53 correlates with alterations in the cellular localization of the tumor suppressor proteins, immunolabeling of p53 protein in T-47D cells was performed followed by laser-scanning confocal microscopy. Consistent with the transcriptional function of this nuclear phosphoprotein, results in Figure 8 reveal that p53 is cytolocalized in the nuclei of T-47D and MCF-7 cells, respectively. This nuclear localization appears predominantly dispersed throughout the nuclear compartment, which can be seen in the DAPI (nuclear counterstain) and p53 merged images. Treatment with E_2_, BPA, and E_2_ + BPA combined showed an increase in the intensity of the nuclear staining of p53 as detected by immunofluorescence. When the cells were exposed to BPA (600 nM), the degree of immunofluorescence was greater than observed in the control (Cs). Those cells treated with BPA + E_2_ combined and E_2_ alone had comparable results, demonstrating the greatest increase in intensity of immunofluorescence. In addition, cells treated with E_2_ + ICI combined and BPA + ICI combined also showed comparable results, demonstrating a lesser degree of immunofluorescence compared to the control. Figure 9 displays the immunolocalization of p53 in MCF-7 cells for comparison. Cells were treated with various combinations of E_2_, BPA, RAL, TAM, and ICI. Figure 9 reveals that the cytolocalization of p53 remains in the nuclei of MCF-7 cells following each treatment condition. The density of nuclear fluorescence correlated well with the protein levels determined by Western blot analysis.

## Discussion

T-47D breast cancer cells express the tumor suppressor protein p53 constitutively.^5,23^ We have previously shown that E_2_ treatment in medium containing charcoal-treated serum causes an increase in p53.^23^ The purpose of this experiment was to study the effects of BPA on the T-47D and MCF-7 breast cancer cell lines and compare the actions of BPA to those of estrogen and other well-known compounds that interact with the ER. Currently, many studies have been conducted only on the MCF-7 cell line, which contains mainly ER, while T-47D breast cancer cells represent both an estrogen and progesterone responsive cell line that expresses protein products of genes for p53.^6,23^ This study was able to compare the two cell lines with similar studies done in each.

The highest levels of p53 expression in T-47D cells were observed upon treatment with 600 nM BPA. Increased concentrations of BPA (1–2 μM) resulted in a decreased level of p53 expression, suggesting that BPA does not have a linear dose dependency. Our findings agree with previous studies performed citing BPA's U-shaped concentration curve.^14^ Optimal concentration for activity appears to be around 600 nM, which produced relative quantities of p53 protein roughly 75% higher compared with the control in T-47D cells. Subsequent cells in the T-47D line treated with 1 and 2 μM of BPA also showed a significant increase in p53 protein levels. In the MCF-7 cell line we also see a U-shaped concentration curve, but with slight variations from the T-47D cell line. In MCF-7, the highest levels of p53 expression are occurring at the 1 μM concentration of BPA.

BPA induced cellular proliferation to the same extent as estradiol (E_2_). In cells treated with E_2_ or BPA, cell counts were 4–4.5 times higher compared with the control. These findings suggest that BPA confers increased cell viability by blocking normal cell mechanisms that induce apoptosis. BPA appears to produce stimulatory cell proliferation in the same manner as estrogen, as both show comparable decreased cell counts when combined with ICI. Such activity at only 600 nM suggests that current levels of environmental human exposure are of concern.^9,11–13,15^ BPA also increased cell viability in a similar manner to E_2_ in the MCF-7 cell line, and the results were reversed when BPA was combined with ICI.

The similar findings surrounding BPA and E_2_ suggest that BPA does indeed have estrogenic activity. BPA and E_2_ increased p53 expression in both T-47D and MCF-7 cells. BPA did so to a lesser degree, most likely due to its slightly lower affinity for the ER.^22,24^ T-47D cells treated with both E_2_ and BPA showed protein levels no higher than cells treated with only E_2_. MCF-7 cells show similar p53 expression levels with treatments E_2_ and E_2_ combined with BPA, with no significant difference between the two treatments. This suggests that no synergistic activity exists when both chemicals are used in combination.

BPAs estrogenic actions were further suggested by its decreased activity in the presence of the pure ER antagonist ICI. While many recent studies have found that BPA does not act through the traditional ER, there is a marked difference in p53 levels between cells treated with BPA and cells treated with BPA + ICI (BPA + ICI cells had half of the relative expression of protein as BPA cells).^22,24,25^ Similar trends are seen in the MCF-7 cell line, which further supports these results. This shows that BPA exerts at least some portion of its effects by binding to ERα and ERβ on cells. If BPA displayed sole utilization of nontraditional ERs, the relative quantity of p53 protein would be nearly equal in cells treated with BPA and BPA + ICI. While cells treated with E_2_ had 1.75 times the protein level of the control, cells treated with E_2_ + ICI had protein levels reduced to levels lower than the control. This same reversal occurred in cells treated with BPA versus cells treated with BPA + ICI.

In addition, there were marked similarities between BPA and E_2_ when interacting with TAM. When cells were treated with TAM in the presence of either E_2_ or BPA, protein levels were increased compared to the control. Both BPA and E_2_ appeared to induce TAMs agonistic abilities. Such findings are of concern, as BPA may be able to lessen the therapeutic benefits of patients using TAM for breast cancer treatment.^14^

**FIG. 10.**
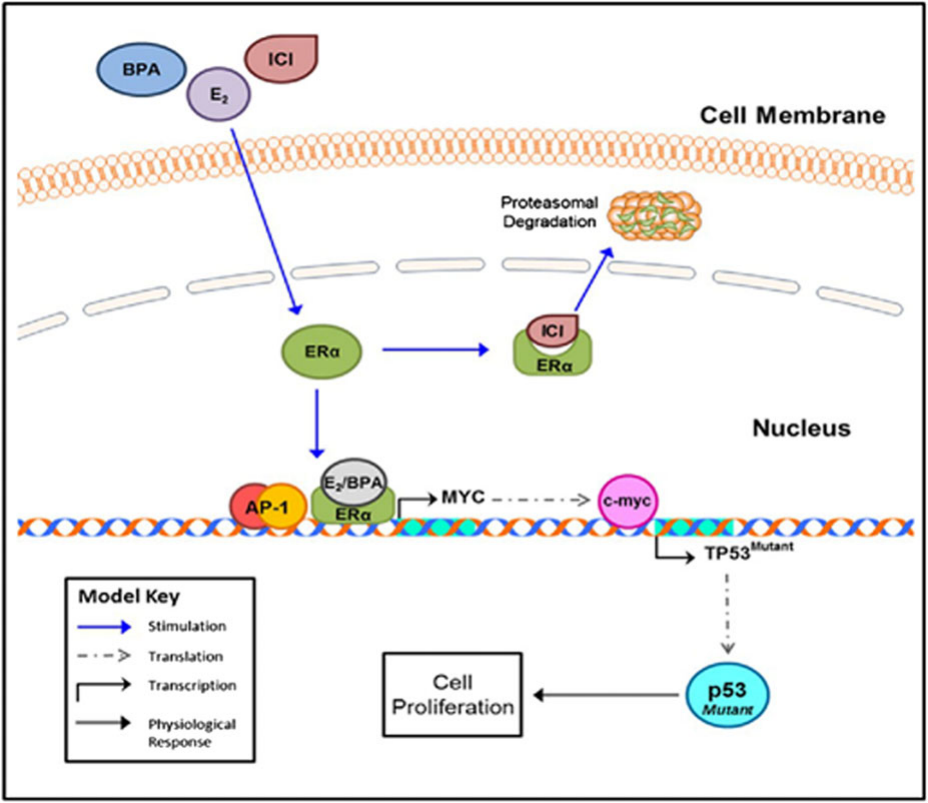
This model shows the potential relationship between E_2_, BPA, and ICI in regulation of p53 and the associated cellular responses.

Our laboratory has previously reported that the actions of p53 are known to be mediated by a product of the p21 gene, which is an inhibitor of cyclin dependent kinases.^7^ This prevents phosphorylation of retinoblastoma protein and, as a consequence, its proliferative effects. The model in Figure 10 describes a hypothetical mechanism of BPA/E_2_/ICI interaction with ERα. Studies have also shown that E_2_ activates the ER through multiple genomic and nongenomic pathways in various tissues and organs.^26^ ERα/specificity protein-dependent activation of E_2_-responsive genes containing GC-rich promoters has been identified in breast and other cancer cell lines. We believe that E_2_ and BPA effects may be mediated using c-Myc gene (Fig. 10), which contains a c-Myc responsive element. It is known that wild-type p53, as expressed in MCF-7 cells, is known to induce DNA repair, apoptosis, or stimulate synthesis of p21 to inhibit cell division. Because T-47D cells contain mostly the mutant form of p53, this may be associated with cell proliferation.

The data presented in this study suggest that BPA has a stimulatory influence on p53 and also promotes cell proliferation in T-47D cells and an increase in cellular viability in MCF-7 cells. Our initial observations suggest that these effects may be ER mediated. Further studies are warranted to clarify correlations between the tumor suppressor genes and ER.

## Abbreviations Used

BPA: bisphenol A
DAPI: 4′,6-diamidino-2-phenylindole
DCC: dextran-coated charcoal
E_2_: 17β-estradiol
ER: estrogen receptor
FBS: fetal bovine serum
HRP: horseradish peroxidase
HSS: high-speed supernatant
ICI: ICI 182,780
MEM: minimum essential medium
NFDM: nonfat dry milk
p53: tumor suppressor protein
pRb: retinoblastoma protein
PBS: phosphate-buffered saline
PMSF: phenylmethanesulfonyl fluoride
PVDF: polyvinylidene fluoride
RAL: Raloxifene
RIPA: Radioimmunoprecipitation assay
SDS-PAGE: sodium dodecyl sulfate–polyacrylamide gel electrophoresis
SSFBS: single stripped FBS
TAM: tamoxifen
TBS: tris-buffered saline
